# Should We Train Alcohol-Dependent Patients to Avoid Alcohol?

**DOI:** 10.3389/fpsyt.2013.00033

**Published:** 2013-05-22

**Authors:** Reinout W. Wiers, Thomas E. Gladwin, Mike Rinck

**Affiliations:** ^1^Addiction, Development, and Psychopathology (Adapt)-lab, Department of Psychology, University of AmsterdamAmsterdam, Netherlands; ^2^Behavior Science Institute (BSI), Clinical Psychology, Radboud University NijmegenNijmegen, Netherlands

Spruyt et al. (2013) report an interesting study in which they compared an alcohol approach-bias, as measured with the Relevant-feature Stimulus Response Compatibility task (R-SRC) in 40 abstaining alcohol-dependent patients and 40 non-dependent controls. While controls had an approach-bias for alcohol as compared to matched control-pictures like water, alcohol-dependent patients showed a relative avoidance bias for alcohol. In the patients group, an avoidance-bias was associated with an increased risk for relapse 3 months later. The authors discuss the relationship of these findings with our training-results, in which alcohol-dependent patients trained to avoid alcohol did better at a 1-year follow-up: “Although initial findings suggest that alcohol-avoidance training could help reduce relapse rates in abstaining alcohol-dependent patients (Wiers et al., 2011), it is still unclear whether changes in automatically approach/avoidance tendencies are directly responsible for the observed changes in treatment outcome. Our findings suggest that actually inducing an avoidance orientation toward alcohol might have harmful effects, at least in a clinical population.”

How can an alcohol-avoidance bias be a predictor of relapse, while alcohol-avoidance training has positive effects in alcohol-dependent patients? Consider two recent clinical alcohol-avoidance training studies. In our first study in 214 alcohol-dependent patients (Wiers et al., 2011), patients trained to avoid alcohol did better at a one year follow-up; in the experimental group, 13% less relapse was found compared with the control groups (either continued assessment or no training). However, as Spruyt et al. rightly point out, the expected mediation of the clinical outcome by a change in approach-bias was not confirmed (as is the case in many studies of Cognitive Bias Modification). In a recent replication study (Eberl et al., 2013), however, mediation was found. In this study, 509 alcohol-dependent patients were randomly assigned to one of two experimental conditions (in addition to “treatment as usual”, as in (Wiers et al., 2011)): they were either trained to avoid alcohol, or received no training at all. Again, training to avoid alcohol had beneficial effects at 1-year follow-up: patients in the experimental group showed 10% less relapse. In this larger sample, the better clinical outcome was mediated by a reduction in approach-bias for alcohol. These data contradict the idea that inducing an increase in avoidance orientations toward alcohol might be harmful in clinical populations: avoidance associations induced by avoidance training were not harmful but beneficial. How can this be reconciled with the data of Spuyt et al.?

First, a correlational relationship between avoidance associations and subsequent relapse does not imply a causal relationship. Perhaps a third variable affected both avoidance associations and relapse probability. Imagine that patients have stronger alcohol-avoidance associations due to more problematic real-life alcohol-approach behavior. Patients with more problematic drinking patterns could also be expected to have relatively high chances of relapse. Another third variable could be differences in (current or previous) interventions, such as pharmacological treatment for alcohol craving: perhaps those patients with higher risk relapse had undergone different treatments that affected their approach/avoidance associations with alcohol. These particular third-variable explanations may or may not be true for the results of Spruyt et al.; they only serve to illustrate that a correlation between avoidance associations and relapse does not imply that the relapse risk was caused by the avoidance associations; and even less that inducing an avoidance association via training would cause an increased relapse risk.

Second, different tasks were used in our studies and the study by Spruyt et al. In their prediction study, Spruyt et al. used the relevant-feature R-SRC, while we used an irrelevant-feature alcohol Approach Avoidance Task to measure the approach bias (alcohol AAT; Wiers et al., 2009) and different varieties of the task to modify the bias. As pointed out by De Houwer (2003), relevant and irrelevant feature tasks are structurally different. Relevant-feature tasks are less implicit in the sense of indirect (participants receive instructions regarding the contents of the stimuli), but are generally more reliable (Field et al., 2011), although reasonably good reliability has also been reported for the AAT (Cousijn et al., 2011). Field et al. (2011) directly compared a relevant- to an irrelevant-feature version of the SRC, and found no correlation between the two measures (*r* = −0.05, *p* = 0.37, Field, personal communication). In our own first re-training study, we also used the relevant-feature approach-avoid Implicit Association Test (Ostafin and Palfai, 2006), and again this measure was entirely unrelated to the AAT (*r* = −0.01, *p* = 0.92). Hence, measuring an approach-bias with a relevant-feature task appears to be unrelated to measuring, and perhaps changing, an approach-bias with an irrelevant-feature task. It could at least theoretically be the case that the kind of avoidance associations as measured by a relevant-feature SRC cause an increased chance of relapse, while alcohol-avoidance associations retrained and assessed by an irrelevant-feature AAT decrease the chance of relapse.

In conclusion, the evidence does not support the idea that the induction of an avoidance bias is likely to be harmful in alcoholic patients: the study of Spruyt et al. does not allow conclusions regarding causality, and a recent training study in fact showed that a relative increase in avoidance mediated the beneficial effects of avoidance training. We do, however, concur with Spruyt et al. that more research is needed regarding the assessment and modification of biases in action tendencies (Watson et al., 2012; Wiers et al., 2013), and other cognitive biases, such as attentional biases, where similar measurement issues arise (Ataya et al., 2012), and where training to avoid alcohol has also shown beneficial effects in alcoholic patients (Schoenmakers et al., 2010). Further, while our replication study showed that a decrease in approach-bias to alcohol stimuli mediates the clinical effect, one could still hypothesize that overly strong avoidance associations may be less desirable than, e.g., more moderate avoidance, a general reduction in salience, or attentional inhibition of distracting or “tempting” stimulus features. Such questions are clearly of potential clinical importance and more research is needed to determine the underlying mechanisms of these novel training interventions.
